# Wabash Valley Æsculapian Society

**Published:** 1870-09

**Authors:** M. W. Wilcox, Wm. E. Henry

**Affiliations:** President; Mattoon, Ill.; Secretary; Mattoon, Ill.


### WABASH VALLEY ÆSCULAPIAN SOCIETY.

MATTOON, ILL., *May 25, 1870.*

Æsculapian Society of the Wabash Valley met pursuant to adjournment; Dr. M. W. Wilcox, President.

The following members were present: Drs. Albin, Dening, Chambers, Johnson, Miller, Massie, St. Clair, Ringland, Swafford, Mosley, Steele, Pearman, Todd, Washburn, Willien, Henry. The minutes of the last meeting, at Paris, Ill., were read and approved.

Dr. Willien's report on surgery was first in order.

This was a paper occupying nearly an hour in its reading, consisting of a statement of cases, their treatment, together with many practical and original ideas. He made mention of the singular phenomenon not usually observed, concerning the cessation of growth in the finger or toe nails in cases of fracture of bones of the arms or legs, which fact it would be well to remember. A case of irreducible strangulated crural hernia of right side, kelotomy, and recovery: Mrs. P——, resident of Effingham, Ill., aged 39 years, mother of five children; hernia of ten years' standing. Was suddenly taken sick in July, 1869, with all the symptoms of strangulated hernia. All ordinary remedies failing, Drs. St. Clair, Lecrone, and he (the operator) decided to operate as the only hope of saving life. The symptons were fast becoming more aggravated, vomiting of stercoraceous matter, etc., etc. *7th instant*—Patient chloroformed; tumor size of 1 rge hen's egg, much inflamed and very painful to the touch. The operation was conducted after the usual manner. Very little blood was lost; hernia reduced. Wound closed with silk thread. Ice applied in after-dressing; opium internally. Suppuration of wound on third day; a simple dressing then of carbolized glycerine and simple cerate. The bowels were moved freely on the seventh by a dose of castor oil. Patient made an immediate recovery, and a radical cure was the result.

The next case was that of peri-uterine abscess; supposed cause, perimetritis. Operation and recovery.

Mrs. H——, aged 27 years, mother of two children. Patient, in act of lifting a tub, was seized with severe pain in back, followed by excessive uterine hemorrhage, with temporary loss of consciousness. She was four months gone in pregnancy, and miscarriage followed.

The pain in back and sacro-lumbar region continued with frequent stranguary, and tenesmus of bowels. The preceding physician in this case, (an irregular), had pronounced the cause of all her trouble a uterine polypus. When the patient desired its removal, he put her under the influence of chloroform and made traction on the neck of the womb; but, wearied out, he declared it would have to be removed by "escoratics." This course of treatment, fortunately, was never attempted.

Dr. Willien saw her first in February, 1869, after an illness of three years. Symptoms: face pale with expressions of anxiety, pulse weak and frequent; chilling; at night a cold and abundant perspiration; pain in abdomen and lower bowels; constipation.

On examination, a tumor size of large infant's head, extending above the pubis about three inches, was painful, fluctuating, and slightly movable. Per vaginal examination: cervex inclined to right, its posterior labia enlarged os partly dilated, and granulated, from which a sero-sanguinous fluid issued of a very offensive character. The uterus was lower in the vagina than normal; distinct fluctuation in cul-de-sac. Per rectum examination: tumor easily defined and fluctuation evident.

Question. Was it an intra-uterine disease, involving its nominal structure, a malignant growth, or a peri-uterine abscess?

Answer. The introduction of the sound showed a declination to the left. The neck was dilated by tent sponge, and uterus found exempt from disease or tumor. He then concluded it to be an accumulation of fluid between the peritoneal covering and tissues proper. Was it serous, blood, or pus? The history of the case led him to think it pus.

*March 30.*—Patient anæsthetized. A small trocar was plunged through the vaginal cul-de-sac, and a rush of white, inodorous pus confirmed his diagnosis. An injection of carbolic acid and rain water was then used, narcotic poultices were applied over the abdomen, and opium internally, to relieve pain. Sulph. quinia and other tonics were freely administered. On fourth day, tumor began to enlarge again, although the discharge continued per vaginum. On examination per rectum, the pressure ruptured the membranes, and a discharge of pus followed the finger. She now began to improve, with a good appetite, and, after a few months, is again about her ordinary domestic work.

Third case was that of fracture of the lower jaw in two places, by kick of horse; one spicula of bone protruding through into the mouth. The interesting part about this case was the application of a new splint, (invented by himself), of vulcanized rubber, made to fit the jaw inside and externally. After six weeks the splints were removed with entire satisfaction.

Dr. Willien, at the close of his report, presented the Society with two new splints for the fore-arm and the leg. These, for their simplicity and cheapness, as well as their real merits, were generally approved. Any physician living near a tinner could have them readily made. A trough-like arrangement, with an expansion for the hand and a perforated septum for the arm or leg to rest on, through which all the superabundant water and effete materials could pass, thence off at one end. A complete dressing could be effected without any disturbance of the limb.

After the reading of this paper, it was discussed by Drs. Swafford, Massie, Chambers, and others.

Dr. Swafford thought the excessive use of cold water by a large part of the profession, and as recommended by some Medical Authors, not a very commendable treatment, many times producing bad results.

Dr. Massie's report on Midwifery was next in order. This document consisted of the Doctor's own views, and how he would conduct labor. It gave evidence of much experience and thought, and was practical and original, written for the benefit of the young men in the profession, and abounded in ideas not to be found in the books. The part referring to ergot elicited debate from some few members, advocates *pro* and *con*, as heretofore.

The report was well received, and requested to be published.

The semi-annual address was delivered by Dr. Swafford, of New Goshen, Ind., and was listened to attentively. It covered much of the history of the profession, from 450 years B. C., down to the merest charlatan or Indian Doctor of the present, showing, with reason, why impostors did and the regular profession did not advertise.

SECOND DAY.—MAY 26.

Society met according to adjournment, at 8 o'clock A.M.

Dr. Chambers, of Charleston, Ill., reported a case:

Mr. C., farmer, age 40 years, June 1st, 1869, was driving into his barn on a load of door-frames; was caught between the load and roof; was carried from his barn to his house, (a short distance). Found on examination, that the last dorsal vertebra was dislocated, one of the ribs on left side fractured, and lower extremities paralyzed. Patient was chloroformed, and extension made, and the luxation reduced. One or two of the processes of the vertebra above were thought to be fractured. A compress with bands about the waist secured the vertebra in place. Morphine was given to relieve the pain. In a few days bromide potass. was commenced and continued in large doses. Urine had to be drawn by catheter. In six weeks strychnia was given, but, not agreeing with the patient, nux vomica was substituted. This remedy seemed to fail, and an electric battery was used for some months.

*May 21, 1870.* Saw Mr. C. to-day; was sitting up in a rocking-chair, in which position he had been some four hours. He weighed, when he received his injury, 160 pounds, was reduced in November last to 100 pounds; now weighs 125 pounds. He presents a healthy appearance, appetite sufficient, tongue clean, skin soft and warm to his toes; can now make partial flexion and extension of the left leg, and slight motion with the right. Sensibility to the knee almost perfect; legs are proportionately smaller than his body; is nearly always admonished when it is necessary to urinate, yet cannot restrain it from passing; bowels rather torpid, thinks he can tell when he is going to have a passage, yet, not always; has had, during the past winter, partial erections of the penis; pulse, in sitting posture 80, recumbent 78.

The case, at this date, is about *statu quo.* It is interesting, from the fact of its being the third on record.

The doctor promised to keep watch over the case, and report to the Society in the fall, as to its progress or condition. He then reported a case of gastrodynia, as cured by sub. nit. bismuth.

A card was then read before the Society, issued by one of its former members:

"Confidential Instructions given to the Married. Dr. Davis has the only reliable and harmless preventive (thereby interdicting the crime of abortion). He also treats successfully, all forms of private diseases, Gonorrhœa, Syphilis, Leucorrhœa, Painful Menstruation, Stricture, Impotence, Seminal Weakness, etc. Call at the —, Room No. —, or address Box 451, Des Moines, Iowa, with 2 three-cent stamps for reply." (Written on card), "$25.00."

He was expelled by a unanimous vote of the Society.

The following gentlemen were assigned as essayists at the Fall meeting:

Dr. A. J. Miller, Paris, Ill., on Baths.

Dr. J. B. Hedges, Clinton, Ind., Obscure Malarial Diseases.

Dr. J. F. Price, Charleston, Ill., Improvements in Therapeutic Agents.

Dr. B. F. Swafford, New Goshen, Ind., Wounds of the Knee-Joint.

Dr. H. H. Deming, Mattoon, Ill., Substitutes for Quinine.

Dr. H. F. Harper, Marion, Ind., Best manner of Building, with a view to Ventillation.

Dr. I. H. Apperson, Filmore, Ill., The Sources of the Gastric Juice.

Dr. J. R. Hinkle, Sullivan, Ind., Spinal Irritation.

The President then appointed the following gentlemen Chairmen of standing committees:

Dr. G. W. Albin, Neoga, Ill., Surgery.

Dr. L. L. Todd, Paris, Ill., Practical Medicine.

Dr. Chas. S. Johnson, New Goshen, Ind., Epidemics.

Dr. E. B. Cannon, Tuscola, Ill., Midwifery.

Dr. R. L. Walstous, Paris, Ill., Indigenous Botany.

Committee of Arrangements for next meeting—Drs. Apperson, Cannon, and Brinton.

Tuscola, Ill., was determined upon as the place for the Fall meeting.

Dr. J. M. Hinkle, of Mattoon, brought up the question of paralysis, in connection with the building of the piers in the Mississippi river, at St. Louis. The workmen having to work in a compressed atmosphere, (under a pressure of about three atmospheres,) were, after a time, (some in a few hours, others after a day or a week) paralyzed. This led him to conclude there was yet a frequent cause for this disease, not fully recognized by the profession. He thought if the external pressure exerted by the atmosphere was a cause of paralysis, as it seemed to be clearly in the above cases, then probably it acted conjointly with other common or recognized causes in every-day life, and his remedy would be to send such cases to more elevated localities, mountainous countries.

The time was so far gone that the debate on this question was cut short, and the Society adjourned to meet the fourth Wednesday in October, 1870.

Dr. M. W. WILCOX, *President.*

Wm. E. Henry, *Secretary.*

